# “This whole thing is relationship medicine”: A qualitative analysis of mobile and street-delivered medication for opioid use disorder in King County, Washington, using RE-AIM

**DOI:** 10.21203/rs.3.rs-9132767/v1

**Published:** 2026-04-01

**Authors:** Marin Strong, Chelsea Rodgers, Kaitlyn Harbick, Kathy Wang, Ohshue S. Gatanaga, Sara N. Glick, Maria Corcorran, Omeid Heidari

**Affiliations:** University of Washington; University of Washington; Public Health – Seattle & King County; University of Washington; University of Washington; Public Health – Seattle & King County; University of Washington; University of Washington

**Keywords:** opioid-related disorders, program evaluation, harm reduction, mobile health units, buprenorphine

## Abstract

**Background:**

Nationally, drug overdose deaths have increased 3-fold over the past two decades, and as of 2023 were significantly higher in Washington (WA) state. Medication for opioid use disorder (MOUD) reduces morbidity and mortality of opioid use; however, delivery often occurs in clinics with multilevel barriers to care. Mobile and street-based models are strategies for engaging individuals at high risk of an overdose by bringing MOUD directly to people who use drugs (PWUD) in the community. This study used the RE-AIM (Reach Effectiveness Adoption Implementation Maintenance) framework to systematically examine cross-programmatic care delivery and sustainability of mobile and street-based MOUD models in King County, WA.

**Methods:**

We conducted semi-structured interviews with frontline providers and administrators between January-June 2025. Eligible programs provided MOUD in King County, WA, and delivered care using a mobile and/or street team model. Using team-based iterative coding, we developed initial codes deductively from REAIM and refined the codebook during consensus coding, allowing inductive codes to emerge. A qualitative descriptive methodology informed by RE-AIM was applied for thematic analysis.

**Results:**

Participants (n = 21) were from 13 unique MOUD programs and were mostly female (57%), held a master’s degree or higher (67%), and had an average of 7 years of work experience with opioid use disorder. Patients reached were often living unhoused with complex comorbidities and were identified through referrals, outreach, and co-location with social services. Effectiveness was often measured by funder-driven metrics that participants felt focused on clinical and process outcomes rather than accurately capturing patient well-being. Flexibility in training, staff roles, practice, and medication protocols was key to the adoption of MOUD in mobile and street environments. Implementation was facilitated by interdisciplinary teams, the combination of established mobile vans with targeted high-touch outreach, and holistic wraparound care. Programmatic maintenance depended on strong community partnerships and diverse funding streams. Sustainability hinged on program capacity meeting increasing demand and on transitioning clients to clinic care, so outreach efforts could continue to center on PWUD with the highest needs.

**Conclusions:**

Programs can leverage these mechanisms to initiate and retain individuals at high risk of opioid overdose with MOUD services.

## Introduction

The rate of drug overdose deaths in the United States has increased greater than 3-fold in the past two decades.^1^ Since 2013, overdoses involving fentanyl and fentanyl analogs have substantially increased.^1^ In 2023, about 70% of drug overdoses nationally involved synthetic opioids like fentanyl.^2,3^ Nationally, drug overdose deaths peaked in 2023 at about 112,000 and fell to less than 70,000 as of 2025 data.^4^ While similar trends were observed in Washington (WA) state in 2023, the rate of drug overdose deaths was significantly higher than the national average – 42.4 per 100,000 people in WA compared to 31.3 nationally.^5^ In King County, WA, there were 1,083 confirmed fatal overdoses from fentanyl in 2023, compared to 167 in 2020.^6^ People who are unstably housed or homeless experience a disproportionate burden of opioid use disorder (OUD) and risk of opioid overdose, and have made up about half of all overdose deaths in Seattle (King County, WA) in 2025.^6,7^

Evidence-based strategies to reduce opioid related deaths include opioid reversal medication (naloxone) and medications for opioid use disorder (MOUD).^8,9^ MOUD, such as buprenorphine and methadone, are highly effective in lowering the risk of morbidity and mortality of opioid use and overdose.^10^ These medications, including long-acting injectable buprenorphine, can substantially reduce opioid craving and withdrawal symptoms.^11^

Although MOUD is highly effective in treating OUD and reducing the risk of overdose, system-level and logistical barriers decrease access.^8,12,13^ Many MOUD delivery models require multiple visits for initiation, have limited clinic hours, and require patients to abstain from opioids.^14^ Additionally, barriers to treatment, including cost, transportation, and availability, hinder initiation.^15^ These limitations result in a majority of people with OUD not being engaged in traditional, clinic-based models of care. In a 2023 survey among King County syringe services program clients, 78% of respondents expressed interest in wanting to stop or reduce opioid use; however, less than half were receiving treatment of any kind.^16^ Only 24% of respondents were receiving treatment with methadone, and 11% were receiving treatment with buprenorphine.^16^

Mobile and street-based models are strategies for engaging individuals at high risk of an overdose by bringing MOUD directly to people who use drugs (PWUD) in the community, overcoming the limitations of traditional clinic-based models. Although implementation depends on context, these models typically provide low-threshold same-day MOUD in non-traditional settings (e.g., parking lots, housing sites/shelters, encampments) using a harm-reduction and flexible approach to care.^17,18^ Evidence on these models remains limited, is largely single program evaluations, and rarely assesses cross-program implementation. Using the RE-AIM framework (Reach, Effectiveness, Adoption, Implementation, Maintenance), which supports the evaluation of programs and evidence-based interventions across settings, we addressed this gap by systematically examining the cross-program care delivery and sustainability of mobile and street-based MOUD models in King County, Washington.

## Methods

### Study design, setting, and population

We employed a qualitative descriptive research design guided by the RE-AIM framework to pragmatically assess how mobile and street-based MOUD models function across programs, given RE-AIM’s emphasis on real-world reach, delivery, and sustainability.^19,20^ We compiled a list of organizations with programs offering MOUD via mobile and/or street team models in the Seattle and surrounding King County, WA, area, using publicly accessible online resources and our knowledge of local providers from practice and research. Programs were eligible for inclusion if they delivered MOUD services using a street team or mobile model. Programs were excluded from eligibility if they provided services exclusively outside King County or if they delivered harm reduction services without OUD treatment. Members of the research team contacted programs via email with a brief study description and attached an informational flyer to initiate engagement and gauge interest. Participants were purposively selected to represent both frontline providers (e.g., registered nurses) and administrators (e.g., medical directors, program coordinators), with the aim of including at least 2 participants from each program to ensure diverse perspectives on programmatic implementation. We also employed snowball sampling, leveraging participants’ networks to identify additional programs that met the inclusion criteria and expand our reach until no new programs were identified. To our knowledge, we identified and contacted all programs that met the inclusion criteria at the time of the study, and only one program did not participate.

### Data collection

A semi-structured interview guide informed by RE-AIM was developed to understand the reach, efficacy, successes, and challenges of implementing mobile and/or street team MOUD programs. The interview guide is available in supplemental materials (Supplemental Table 1). It was piloted and refined in the first two interviews. Following recruitment, eligible participants were scheduled for an interview and sent an electronic survey to capture demographic and program data.

MS, a registered nurse and doctoral student who has been trained in qualitative research methods and study protocols, scheduled and conducted all interviews. OH, an experienced MOUD clinician and qualitative researcher, was present for the first two interviews to ensure high-quality data collection. Co-authors (MC, clinician-scientist; SG, epidemiologist) have extensive experience with MOUD, low-barrier care models, and working with marginalized and unhoused populations, and were consulted throughout the study design, data collection, and data analysis.

Participants (e.g., program employees, not patients) were interviewed via Zoom or Google Voice to maximize flexibility; all declined in-person offers. All participants provided verbal informed consent and were compensated with a $50 electronic gift card for their time. Interviews lasted between 40 and 70 minutes. Interviews were audio-recorded and transcribed verbatim using RevAI, an AI-powered speech-to-text service.^21^ All transcripts were human-checked for accuracy and completeness. All data was securely managed in a REDCap database.

The Institutional Review Board of the University of Washington reviewed this study and determined it to be human subjects research that qualifies for exempt status (STUDY00021749).

### Data analysis

A team-based, iterative coding approach was used. The study team deductively developed initial parent and child codes from the interview guide and the RE-AIM framework. Next, five team members (MS, KH, KW, CR, OG) independently blind-coded two transcripts and met virtually to reconcile coding differences and refine code definitions through an iterative process. During these sessions, additional inductive codes were identified, discussed, and defined. A third transcript was used for consensus coding, and the codebook was finalized when confirmability and consistency across coders were reached. All transcripts were subsequently double-coded. Primary coding was completed by four team members (KH, KW, CR, and OG), and secondary coding was performed by a single coder (MS) to ensure accuracy and consistency. Dedoose version 10.0.59 was used to manage all data and coding. Data were analyzed thematically within the RE-AIM framework. The Consolidated Criteria for Reporting Qualitative Research guided data collection, analysis, and reporting.^22^

## Results

From January to June 2025, we conducted 21 interviews with staff from 13 programs that provide mobile and street-based MOUD in King County, WA. While we attempted to interview one administrator and one frontline provider from each program, a few programs had just one participant each. Participants were mostly female (57%) and had an average age of 41 years (range: 26–62 years). Nearly all participants had a bachelor’s degree or higher (95%), and a plurality held a doctorate degree (38%). Participants reported a range of 16 months to 20 years of total work experience with opioid use disorder, harm reduction, and/or mental health services (median: 5.5 years) and a range of 3 months to 13 years (median: 2.75 years) in their current role. Participants represented the diversity of mobile and street teams, including direct care roles (e.g., nurses) and administrative roles (e.g., medical directors). Participants’ additional certifications, licenses, and training were in wound care, bioethics, mental health, buprenorphine prescribing, addiction medicine, emergency response, public health, family medicine, and Drug Enforcement Administration (DEA) licensing. A summary of participant characteristics is available in Table 1.

Participants were from 13 unique MOUD programs that deliver services via mobile and/or street-based teams. The non-clinic settings of these programs ranged from EMS and fire department teams that initiate buprenorphine post-overdose and during field outreach, to providers integrated into supportive housing and tiny home communities, to case-based outreach across carceral and community settings. Mobile vans were the most common (9 of 13 programs), with some operating at fixed locations and others traveling to multiple locations to provide care. Some programs had been operational for over 10 years, while others had been operational for only a few months, providing rich depth for evaluating implementation across many stages. Further details on services provided by programs can be found in Table 2.

Thematic findings, organized according to the RE-AIM domains of Reach, Effectiveness, Adoption, Implementation, and Maintenance, are described below and summarized in conclusion in Table 4.

### Reach

Programs identified patients through a diverse mix of referrals, outreach, and co-located services. Community partners frequently requested assistance from programs through informal collaboration or formal agreements, while referrals to programs came from friends and family, especially when the referrer was engaged in mobile or street care themselves. Referrals were also internal to programs’ parent organizations, as they sought to link care between the clinic and mobile or street settings to accommodate patients’ lifestyles and needs, such as when a street team connects a patient living unhoused for MOUD planning prior to discharge:

“We could take referrals from inpatient teams for their folks that they know are going to be discharged, unsheltered and unhoused for us to go actually meet them when they’re in the hospital and then introduce ourselves, offer services, and try to find them when they’re discharged.”

– R15, medical doctor, street team

Patients were described as not well engaged in clinic-based care, often living unhoused or in supportive housing, and navigating competing daily demands that limited their ability to engage. Patients were often transient and engaged with different programs and services throughout the Seattle and King County area based on their needs and location. In contrast, participants noted a patient subpopulation that were stably engaged in MOUD care for years, but preferred delivery via mobile van models. Difficult-to-reach populations included people without insurance, migrants, non-English speakers, those with limited mobility, and patients living outside the city center.

“We have a lot of people who don’t engage in any kind of routine care…that may be because they’re going in and out of their addiction treatment…A lot of my patients there are not housed, have mental health comorbidities, have a lot of stress, and kind of chaos in their lives. And so they may just not be in a place where it’s a priority for them to really engage in what we would classically think of as primary care.”

– R22, medical doctor, medical director, mobile team

Within King County, the geographic spread of programs was concentrated around Seattle’s city center, driven by need and accessibility, but services were also intentionally located in the northern and southern areas of King County to increase access where there are otherwise few to no other MOUD providers. Some programs are geographically restricted to King County due to staff availability or funding mechanisms, while others operate mobile vans throughout the broader area. Outreach occurred through structured systems, such as scheduled days at designated mobile van parking spots, “anchor sites”, and targeted outreach to areas of known drug use. Many mobile van programs dedicated staff time for monitoring and periodically canvassing the surrounding areas to engage people who might not approach the van. Street-based teams were best positioned to leverage outreach as a means of engagement and were most responsive and flexible in accommodating resource-intensive individual outreach:

“…it’s really, really important that we continue to get out of the car and go to the tents because oftentimes the folks that can’t or have a really hard time engaging are not going to be the ones coming to ask for [care].”

- R24, nurse practitioner, street & mobile teams

### Effectiveness

Programs tracked a diverse and dynamic set of engagement, clinical, and systems-level metrics to monitor effectiveness. Common metrics included the number of visits, new and unique patients, return and follow-up visits, retention in care, MOUD initiation and dosing (e.g., injection timing and prescription refills), demographic characteristics, housing status, and harm reduction supply distribution. Some outcomes focused on utilization, including emergency department visits and diversions, jail stays, and 911 call volumes. Programs also monitored referrals and linkages to housing and primary care, conditions treated and services provided, and adverse outcomes.

In contrast, participants critiqued these measures of effectiveness, noting that the metrics often failed to accurately reflect the true impact of their work. Most definitions of effective care and service delivery were rooted in clinic-based settings, and participants preferred to shift the focus to patient well-being and quality of life. Some administrators noted that they had data available and/or a vision for improved metrics but lacked the resources and support to build the necessary systems. Other participants commented on the faulty logic behind some metrics, such as the utility of avoided ER visits, when, at times, that is a desired outcome. Participants also noted the difficulty of managing reporting across multiple funding sources, with little to no harmonization of metrics, leading to disparate data collection. Many programs sought to collect qualitative data from patients to understand whether their needs were being addressed:

“we are…sort of like a mobile social work team. And social work is really hard to capture quantitatively. And if I had three extra full-time employees just like coding and entering, I’m sure I could figure out a way, but I don’t, it’s all just on the practitioners themselves.”

– R19, program manager, mobile + outreach teams

Participants frequently defined success on their own terms as continued engagement. Having a patient return to the van or speak with the street team was a win, regardless of health outcomes or services. Other patient-centric ideas of success included patient-driven goal making, patient satisfaction, improved quality of day-to-day social life and well-being, and fostering a safe environment for patients. Some participants conceptualized success at the team level, defining it as collaboration, improved skills, and a sense that they have the necessary resources and tools to meet patient needs. Participants also spoke about adjusting their perceptions of success as they began engaging in mobile and street-based MOUD, shifting away from clinical outcomes and moving toward building trust:

“The population we serve is extremely marginalized. They get a rough go of it…The fact that they make a conscious choice to come back every day to get help, to get treatment, to take steps to [make] their life better, making themselves feel better. That’s how I define success.”

- R17, program manager, mobile team

### Adoption

Programs adopted MOUD service delivery to mobile vans and street-based settings by leveraging multipurpose training, adapting resources to field environments, and embracing flexible practice and protocols. Training staff for field-based delivery emphasized competencies in de-escalation, trauma-informed care, and OUD/MOUD-specific skills, including handling of controlled medications and conducting induction protocols. Training often evolved in response to patient needs, including wound care and expanding clinical skills to address care gaps in the field (e.g., sexual health). Many programs used shadowing and routine team meetings to provide skills exposure and foster open dialogue about up-to-date practices. Task-shifting and cross-training were common, improved care efficiency, and were supported by adapted protocols.

Resources necessary for care delivery were adapted to the unique mobile or street-based settings. Programs that stored medications with partner agencies or on mobile units had to adopt spaces for MOUD dosing, including medication pumps, secure windows, and locked cabinets. Mobile units required maintenance plans, such as grey-water management, parking permits, and consideration of weight and size restrictions during transit. For documenting and delivering care, participants noted that technology sometimes constrained care delivery, such as spotty internet access in the city, but it also aided care, including specialized EHR ordering sets for field-delivered MOUD and wireless temperature regulation. Street teams were equipped with neon backpacks to increase visibility and carried items like cigarettes to encourage patient interaction. These program staff also discussed preparation for a variety of care situations at a moment’s notice in the community:

“we always have an emergency bag with us just in case we have responded to opioid overdoses…we’d like to be prepared with lifesaving equipment while we’re calling 911…We always bring the engagement supplies. I mentioned harm reduction, but also basic need supplies like water and snacks…We all have these big backpacks and the nurses in their packs carry wound care supplies, a bunch of point of care tests…And the provider bags is essentially the pharmacy.”

– R24, nurse practitioner, street & mobile teams

Participants discussed how MOUD delivery was adopted for the field by leveraging flexible practices and protocols that were regularly reviewed and updated in response to patient needs, logistical factors, and emerging practice evidence. Examples included expanded pharmacy partnerships to improve MOUD access; delivering MOUD directly to patients; brief provider telehealth consultations to facilitate sustainable billing; patient transportation to and from mobile sites; provision of limited suboxone films to stave off precipitated withdrawal; and eliminating appointments. A key innovation was a 3-day injectable-only overlapping buprenorphine starting method that resulted from programs iterating in response to patient feedback:

“… that induction packet took about 14 months to get to where it’s at, and that went through many forms of trying some different inductions with people and being creative, because we knew that the on-label options didn’t work, in fact were terrible for the population of people using fentanyl. So we made some adjustments… It requires some flexibility in nursing, it requires flexibility with a provider…the larger story here…is what it took to get to the protocol and the flexibility required to provide treatment to a population that just does not respond to standard treatments and methods.”

– R5, registered nurse, case manager, mobile team

### Implementation

Programmatic implementation was grounded in interdisciplinary teamwork and informed by a confluence of factors, including mission, funding, target population, and parent organization. A summary of each program is presented in Table 3. Strengths across programs included the combination of established mobile vans serving broader populations, alongside newer, more targeted interventions (e.g., post-overdose response and intensive case management for highly justice-involved individuals). Programs were hindered by a lack of weekend coverage, limited geographic spread, and an overall mismatch between current capacity and increasing demand for MOUD.

Direct implementation roles commonly included case managers, outreach specialists, drivers, nurses, social workers, peer navigators, licensed prescribers, and medical assistants. Indirect support roles included IT professionals, pharmacists, medical directors, clinic staff, and folks working at community partners, like case managers at supportive housing, who aided program staff on-site. Team size ranged from 1–2 people on 1:1 outreach-based teams to larger 6–10-person teams on an established mobile unit. Street teams were typically 2–5 people. Interdisciplinary teams were essential to implementation, with each role contributing unique skills and perspectives, especially that of program staff with lived experience:

“I think it’s the team that you assemble…We are a second chance employer. A lot of our staff come from a place of lived experience…and that you can identify with and connect with patients on a very different level…that keeps them engaged with us.”

– R17, program manager, mobile team

Participants described patient interactions as time-sensitive touchpoints to do as much as possible; thus, programs aimed to provide holistic, wraparound care whenever they had the capacity to do so. Psychiatric care was typically offered as an interim solution for patients not established with mental health providers; as one participant called it, “very light behavioral health.” Only two programs consistently had psychiatric specialists on their teams, and while social workers and case managers tried to refer to local behavioral health resources, they were limited by availability. Patients were given food, drinks, clothing, hygiene kits, cold-weather gear, tarps, and tents. Referrals to and coordination with services such as transportation, housing, clinic-based primary care, and rehabilitation were essential to facilitating client engagement. These services often helped to establish a trusting relationship upon which future, more effective MOUD conversations could be had. Patient engagement was further facilitated by distributing harm-reduction supplies, partnering with trusted community organizations, creating a non-stigmatizing care environment, maintaining a consistent, reliable presence, and prioritizing patient autonomy and agency in their care.

“There’s a group of people that it’s very slow but steady. It’s like planting seeds; sometimes it’s just getting people a new pair of clothing and an Ensure and harm reduction supplies for weeks until they can trust us enough to come in for a more formal clinical visit.”

– R18, registered nurse, street + outreach team

### Maintenance

The sustainability of mobile and street-based MOUD programs depended on dedicated staff who built strong community partnerships, pursued diverse funding sources, and aligned program priorities with those of their parent organizations. At the time of this research, participants viewed ongoing federal investment in substance use, mental health, and homeless services as essential to the maintenance of these efforts. Strong relationships were crucial for sustaining the work and were built on shared mission-driven goals and alignment among programs and community partners. Participants commented on the difficulty of advocating for funding for their specific program, given the knowledge that they may not see or be able to clearly measure a return on investment; rather, the overall system will. Some programs found sustainability in balancing these demands by aligning the work and funding of street and mobile teams with clinic-based care:

“If I was only seeing people in the streets of [city], we wouldn’t be making money, but seeing people in the streets of [city] while prioritizing our pharmacy to deliver medicine, which also brings in a lot of money for our organization, while also keeping our clinic open to be able to see people in the clinic while a team is out”

– R11, medical doctor, medical director, street + outreach team

Participants noted that balancing the increased demand driven by the success of the 3-day injectable-only buprenorphine induction with maintaining sustainable patient engagement was key to the continued success. Narratives emphasized the need for ongoing outreach to engage patients not currently served by clinic models, while also recognizing the importance of transitioning patients to clinic care when possible. Clinic care was perceived as more efficient, allowing resource-intensive outreach to focus on reaching the most difficult-to-engage individuals.

“when we pause and really reflect on the numbers… we realize that we’ve hit our capacity…if we’re really aiming for solid retention and care, that does limit the number of new people we can accept. And so we are having conversations about the idea of imposing eligibility criteria, trying to identify the target population that we think we are most suited to reach and trying to refer everyone else to other existing brick-and-mortar clinics.”

– R20, medical doctor, medical director, mobile team

Funding for programs is complex, fragmented, and often unstable. Grants were frequently a starting point, allowing programs to trial the mobile model without constraints. However, as they transitioned to long-term implementation, programs often struggled to cover the high operational costs of field-based work. Exacerbating this was the fact that reimbursement structures typically pay per MOUD dose and per provider visit, failing to cover the costs of outreach, interdisciplinary care, transportation, and other wraparound services - all of which are essential to the delivery model. Injectable MOUD was highly effective but expensive, requiring Medicaid billing for appropriate reimbursement and leaving uninsured patients with gaps in care because of limited staff capacity to provide one-to-one insurance navigation. To address this, programs advocated for state-level changes, such as buy-and-bill drug programs, and, when possible, integrated provider consultation into street-based care – though not without cost:

“Most fee-for-service hinges on the provider…so you have an incentive to have a provider see a patient, even if that provider perhaps could be doing something more meaningful, because the nurse is well capable…You start allocating your provider resources to try and spread the provider thinly so they’re touching many different patients, which in some ways can burn out a provider, maybe make a nurse feel a little less empowered and yeah, just distorts where you put resources.”

– R20, medical doctor, medical director, mobile team

Some participants expressed frustration that programs have to compete for funding, especially given that most share the same goals and serve a similar population, and wished for more communication and collaboration among programs. Participants noted a tension between the long-term system savings of providing MOUD to people with the highest needs, who are often the highest utilizers of care, and the high upfront costs of operating field-based teams, including staffing, logistics, mobile units, and maintenance.

“…setting up field-based teams is labor intensive and time intensive…and it’s a fantastic model of care. It’s also not without cost. I think ultimately [it] could be cost effective to the system, but the person paying is often not the person benefitting.”

– R21, medical doctor, medical director, street + outreach teams

## Discussion

This study explored the cross-programmatic characteristics of mobile and street-based MOUD models in King County, Washington, through the lens of RE-AIM. We found that mobile and street-based MOUD models primarily served PWUD experiencing complex comorbidities, social instability, and homelessness. Programs reached patients through structured outreach, multiple referral streams, and intentional partnering with social services. Measures of effectiveness were often disparate, driven by funders, and insufficient for assessing the impact of relational, social care, and foundational to the model. Dynamic training, flexible staffing, and adaptable protocols were essential to the adoption of MOUD in mobile and street settings. Successful implementation was aided by interdisciplinary teams, comprehensive wraparound support, and collaboration between mobile infrastructure (e.g., vans) and high-touch one-to-one outreach. Sustainability was supported by strong community partnerships and the resilience of diversified funding sources. Long-term viability depended on balancing rising MOUD demand with pathways that transition clients to clinic-based care, so that mobile and street-based models can remain focused on engaging PWUD with the highest unmet needs.

Mobile and street MOUD programs in this study demonstrated extensive reach to marginalized and vulnerable individuals (e.g., sex workers, people immediately post-overdose, highly justice-involved individuals), aligning with prior literature that these models expand access for underserved and high-risk populations.^10,17,23,24^ Where most studies examine single programs in urban or rural settings, this study sampled a wide geographic area, allowing us to reflect on the concentration of programs around the high-need urban center of Seattle and identify periurban gaps in MOUD access that could be addressed by expanding mobile services. Further, mobile MOUD models generally reduced, but did not eliminate, transportation barriers across settings, leading several programs in this study to offer rides to and from mobile sites, a strategy used by mobile MOUD models in Chicago and Oregon.^18,25^ In practice, both this study and a review of programs across the US identify the same pattern: although patients served by these models frequently have complex psychiatric comorbidities and programs offer mental health support, very few explicitly provide robust psychiatric care.^26^ This highlights how rare integrated behavioral health services remain within mobile and street-based MOUD models and underscores the opportunity to move beyond referral-only or ad hoc approaches.

Adoption hinged on multipurpose training (de-escalation, trauma-informed care, OUD/MOUD skills), task-shifting, and iterative protocol development. Expanding on established collaborative practice and holistic care models, interdisciplinary teams leveraged peers and staff with lived experience to build trust, and nurse-led models often bundled MOUD with wound care, infectious disease services, and tangible supports (food, clothing, harm-reduction supplies).^18,23,23,26–28^ A notable practice innovation started by one site was a 3-day, injectable-only overlapping buprenorphine start protocol, developed through iterative learning to mitigate precipitated withdrawal among people using fentanyl.^29^ This highly effective protocol and its swift adoption by multiple mobile and street-delivered programs in this study often leveraged nurse-led initiation and retention, which was aided by peers in building trusting relationships. This study identified a clear disconnect between the programmatic and funder-driven metrics commonly used and participants’ views that these measures fail to capture the true, patient-centered success of their work. Similar misalignment has been documented in MOUD programs, where patients prioritize emotional well-being, functioning, and individualized goals over traditional clinical indicators.^30^ Syringe service programs face a similar dynamic, with external reporting requirements often diverging from client-centered, harm-reduction priorities.^31^ Program sustainability relied on strong community partnerships, diversified funding sources, and alignment with parent organization priorities. As demand grew, frequently driven by the 3-day injectable-only overlapping start method, programs emphasized the need to transition patients into clinic-based care so outreach teams could focus on patients with the highest unmet needs. Long-term viability remained challenged by fragmented funding, high operational costs, and reimbursement models that fail to cover the intensive, interdisciplinary work required for mobile and street-based MOUD delivery. A recent narrative review identified similar barriers across 41 mobile addiction treatment programs, including insufficient reimbursement, regulatory constraints on buprenorphine, high costs, and lack of patient insurance.^26^

Strengths of these findings include a robust sample of participants working in direct care and in programmatic management of mobile and street-based teams, which facilitated multi-level and multi-perspective insights. The programs included in this study were at varying stages of implementation and employed diverse strategies, providing rich contextual insight and opportunities for cross-program interpretation. The limitations of this work include: (1) its geographic restriction to King County, WA is not generalizable to all U.S. contexts due to the specificity of the local innovation environment and the local and state regulations that enable these programs; (2) There is potential for bias, as many team members work professionally in the MOUD and low-barrier spaces; and (3) There is a distinct lack of perspectives from other priority groups in MOUD delivery, including patients and funders.

## Conclusion

Mobile and street-based MOUD are critical complements to brick-and-mortar care, improving reach and engagement of populations at the highest overdose risk through relationship-based care. Sustained impact will require harmonized metrics that reflect patient priorities and reimbursement models that reflect the increased level and diversity of services needed to engage priority populations in treatment. Findings from this study inform the characteristics of successful implementation of these care delivery models, gleaned across various programs. Future research should examine models of mobile and street-based MOUD in non-urban settings and other geographies, using longitudinal data, and test the cost-effectiveness of field-based treatment models, particularly when providing care with low-barrier principles. Addressing these gaps is critical to scale-up and adoption, while regulatory frameworks must also evolve to support the implementation of street- and mobile MOUD models, including policies that improve field-based medication access and buy-and-bill mechanisms for MOUD that improve efficiency and timeliness of MOUD delivery.

## Supplementary Material

Supplementary Files

This is a list of supplementary files associated with this preprint. Click to download.

- SupplementalMaterials.docx

## Figures and Tables

**Table 1:** Demographics of front-line providers and administrators of programs delivering MOUD via mobile and street-based models in King County, WA (N=21)

	n	% or std. dev.
Gender identity		
Female	12	57%
Male	7	33%
Nonbinary and/or genderqueer	2	10%
Mean age (std. dev.)	41	11
Highest education		
Doctorate (MD*, PhD^†^, DNP^◊^)	8	38%
Masters	6	29%
Bachelors	6	29%
GED^∞^	1	5%
Mean years of total work experience with OUD, mental health, and/or harm reduction (std. dev.)	7	0.71
Mean years in current role (std. dev)	3.9	3.2
Role/license		
Registered nurse	5	24%
Advanced registered nurse practitioner	3	14%
Physician	6	29%
Project manager/program coordinator	4	19%
Physician associate	1	5%
Medical assistant	1	5%
EMT^#^	1	5%

* MD: Doctor of Medicine;

† PhD: Doctor of Philosophy;

◊ DNP: Doctor of Nursing Practice;

∞ GED: General Education Diploma;

# EMT: Emergency Medical Technician

**Table 2:** Services provided by programs delivering MOUD via mobile and street-based models in King County, WA (N = 13)

Service Type	Total Provided		Location provided (n)		
	*n*	*%*	Community (non-clinic)	Mobile	Community + mobile
Naloxone	13	100%	4	4	5
Overdose education	13	100%	5	2	6
MOUD	13	100%	4	4	5
Counseling for OUD	13	100%	4	3	6
Referrals for OUD treatment	12	92%	4	2	6
Addiction and chemical dependency counseling	8	62%	2	2	4
Distribution of sterile drug use supplies	8	62%	5	0	3
Infectious disease testing	9	69%	3	2	4
Infectious disease treatment	10	77%	4	2	4
Wound care	9	69%	4	0	5
Housing assessment and referrals	6	46%	2	2	2
Counseling for mental health and behavioral health	7	54%	3	1	3
Medication for mental health and behavioral health	10	77%	2	2	4
Referrals for mental health and behavioral health	10	77%	4	1	6

**Table 3:** Implementation summary of mobile and street-based MOUD programs in King County, WA (N = 13)

Program	Modality	Target population	Timing of engagement	Primary MOUD	Unique characteristics
1	Mobile unit + 1:1 Outreach	People post-overdose	Day of overdose; 1–3 day touchpoint; multi-week follow-up as needed	Buprenorphine (SL*)	Embedded in emergency response Paramedic & EMT administered MOUD
2	Mobile unit	PWUD^◊^	Weekly, monthly	Buprenorphine (SL), methadone, naltrexone	Large RV-style mobile clinic
3	Mobile van	Women with a lifetime history of injection drug use	Daily for 12 months	Buprenorphine (SL)	Research pilot project
4	Mobile van	PWUD	Daily (weekdays)	Buprenorphine (LAI, SL^†^)	3-day injectable only overlapping start method
5	Mobile van	PWUD	Daily (weekdays)	Buprenorphine (SL), methadone, naltrexone	
6	Mobile van	Tiny home communities	Weekly, monthly	Buprenorphine (SL)	Health science student training
7	Mobile van	PWUD	Daily (weekdays), weekly	Methadone, buprenorphine (SL)	Dosing only vans + off-site provider consultation MOUD delivery to medical housing
8	Street outreach + 1:1 outreach	PWUD	As needed, episodic	Buprenorphine (LAI, SL)	High flexibility RNs bridging care between clinic & community
9	Street outreach	PWUD, immediate post-discharge from hospital	Weekends, episodic	Buprenorphine (SL)	Inreach to patients while still admitted in hospital Weekend coverage
10	1:1 outreach	Highly justice-involved individuals with psychiatric disorders + OUD	As needed; daily, weekly, monthly	Buprenorphine (SL)	Inreach to patients in jail Nurses as legal system advocates Long term engagement
11	Street outreach	PWUD	Daily (weekdays)	Buprenorphine (SL)	Equity-based patient engagement metrics “anchor sites” + outreach
12	Street outreach	PWUD	Daily (weekdays)	Buprenorphine (SL, LAI)	
13	Mobile van	PWUD	Most weekdays; as needed	Buprenorphine (SL)	Telemedicine

* SL=sublingual;

† LAI = long-acting injectable;

◊ PWUD = people who use drugs

**Table 4:** RE-AIM thematic findings

RE-AIM	Description	Illustrative Quotations
**Reach** *How programs identify, reach, and engage with clients, and which populations are typically served*	Patients reached were often living unhoused with complex comorbidities and were identified through referrals, outreach, and co-location with social services.	“I am fielding calls every day from different areas in the community that ask…can your [mobile van] come out? Can you guys come out? I mean, the community need is so intense.” - *R17, program manager, mobile team* “I’m seeing there’s still a population of people who aren’t accessing traditional healthcare, maybe going to the ER when things are really, really bad… [for] different reasons: stigma, people can’t leave their stuff unattended, people are feeling really sick and they can’t get in.” - *R2, registered nurse, mobile team*
**Effectiveness** *How programs evaluate delivery of MOUD and care, and ensure the efficacy of their services*	Effectiveness was often measured by funder-driven metrics that participants felt focused on clinical and process outcomes rather than accurately capturing patient well-being.	“It is inefficient, from a volume perspective, and yet it’s highly efficient from a “reaching the people that aren’t being engaged” perspective…If you have a nurse going to do injections in the field…. maybe they get five or eight injections in a day….And someone….in a fast-paced primary care clinic might look at that and say…we could do 25…but you’re not reaching the same people. We think sometimes funders or reimbursement models can miss that.” – *R20, medical doctor, medical director, mobile team*
**Adoption** *How programs adopt protocols and practices to mobile and street-based settings, and the required training, skills, and resources to do so*	Flexibility in training, staff roles, and practice, along with medication protocols responsive to patient needs, were key to the adoption of MOUD in mobile and street environments.	“They’re working to cross-train…so that the driver can do a little bit more of the front desk work. The front desk person can do more of the MA work. The MA can do more of the front desk work just so that there is a little bit more overlap. Because oftentimes with that small of a team, if somebody has a task that’s taking up a lot of time, it produces a bottleneck…” – *R10, physician’s associate, mobile team*
**Implementation** *How programs deliver MOUD and the types of services provided*	Implementation was facilitated by interdisciplinary teams, the combination of established mobile vans, targeted high-touch outreach, and holistic wraparound care.	“you can provide more wraparound, interdisciplinary, interprofessional care to patients. There’s no one team member who can do everything. And so having people with whom you can readily collaborate and communicate provides better care for patients who are particularly vulnerable and need the best care.” - *R21, medical doctor, medical director, street + outreach teams* “trying to use those moments… [as] a window to bring up broader harm reduction conversation, bring up MOUD…at every turn trying to just mention what we can offer…trying to solicit what they want and what they need. And then trying really hard to, in the moment..to satisfy this need if we can…chances are we’re not looking [for] the person again, or at least not intentionally, because places get swept all the time or people move around so much.” – *R16, registered nurse, street team*
**Maintenance** *How programs sustain services*	Maintenance depended on strong community partnerships and diverse funding streams. Sustainability hinged on program capacity meeting increasing demand and on transitioning clients to clinic care, so outreach efforts could continue to center on PWUD with the highest needs.	“the reason why the team is maintained is because the work is meaningful, and the work is meaningful because we’re actually seeing results for people. I think we’re seeing results because of these partnerships that we have.” – *R11, medical doctor, medical director, street + outreach team* “It’s not coming through a paid healthcare model. It’s not coming through a private sector approach. I think here, the public sector model…really makes a lot of sense and it really allows us to be genuinely low barrier, which is the key.” – *R19, program manager, mobile + outreach teams*

## Data Availability

The data analyzed during the current study are available from the corresponding author on reasonable request.

## Abbreviations

WA: Washington
MOUD: Medication for opioid use disorder
OUD: Opioid use disorder
DEA: Drug Enforcement Agency
MD: Medical doctor
PhD: Doctor of Philosophy
GED: General education diploma
RE-AIM: Reach, Effectiveness, Adoption, Implementation, Maintenance
DNP: Doctorate of Nursing Practice
EMT: Emergency Medical Technician
EMS: Emergency medical services
PWUD: People who use drugs
SL: sublingual
LAI: long-acting injectable
